# Population-based ultrasound prevalence and risk factors for cystic echinococcosis in endemic Kazakhstan

**DOI:** 10.1371/journal.pntd.0014126

**Published:** 2026-03-16

**Authors:** Gulziya Ismailova, Majid Fasihi Harandi, Shokan Kaniyev, Zhanna Shapiyeva, Daniyar Mukazhanov, Bolatbek Baimakhanov, Adriano Casulli

**Affiliations:** 1 JSC Syzganov National Scientific Center of Surgery, Almaty, Kazakhstan; 2 Al-Farabi Kazakh National University, Almaty, Kazakhstan; 3 Research Center for Hydatid Disease in Iran, Kerman University of Medical Sciences, Kerman, Iran; 4 Department of Medical Parasitology, School of Medicine, Kerman University of Medical Sciences, Kerman, Iran; 5 Scientific and Practical Centre for Sanitary and Epidemiological Expertise and Monitoring, Almaty, Kazakhstan; 6 WHO Collaborating Centre for the Epidemiology, Detection and Control of Cystic and Alveolar Echinococcosis (One Health), Department of Infectious Diseases, Istituto Superiore di Sanità, Rome, Italy; 7 European Union Reference Laboratory for Parasites (EURL-P; food safety), Department of Infectious Diseases, Istituto Superiore di Sanità, Rome, Italy; 8 European Union Reference Laboratory for Public Health on Helminths and Protozoa (EURL-PH-HP), Department of Infectious Diseases, Istituto Superiore di Sanità, Rome, Italy; University of Zurich: Universitat Zurich, SWITZERLAND

## Abstract

**Background:**

Cystic echinococcosis (CE), caused by *Echinococcus granulosus sensu lato*, remains a significant public health concern in endemic areas of Kazakhstan. Despite global control efforts, CE persists due to insufficient deworming of dogs, the absence of control programs, and low public awareness.

**Methods:**

A cross-sectional study was conducted between September 2023 and June 2024 in 51 remote villages across two endemic regions of Kazakhstan. Participants underwent abdominal ultrasound screening and completed a structured questionnaire to assess potential risk factors. Cyst staging was performed according to the WHO - Informal Working Group on Echinococcosis guidelines. Statistical analyses included both univariate and multivariate models to identify significant predictors of infection.

**Results:**

The prevalence of CE was 0.34%, with higher rates observed in young people (82.1%) than in adults (17.9%), particularly among those aged 9–14 years. Major potential risk factors included dog ownership (OR = 3.17, p = 0.012), failure to deworm dogs (OR = 11.12, p = 0.018), feeding raw offal to dogs (OR = 3.06, p = 0.012), and consumption of unwashed vegetables and fruits among women (OR = 5.25, p = 0.005). All identified CE cases were newly diagnosed. Of these, 92.85% were active cysts, predominantly found in young individuals, while inactive cysts accounted for 7.14%, distributed equally between adults and youths.

**Conclusions:**

These findings underscore the urgent need for regular deworming programs for dogs, public education on hygiene practices, and stricter management of livestock offal. Further research is needed to investigate transmission through water and environmental contamination.

## Author summary

Cystic echinococcosis (CE) is a dog-transmitted parasitic disease common in rural communities where livestock farming is practiced. In Kazakhstan, this disease remains a major public health problem, yet its true burden has been poorly understood. We visited 51 villages, screened 8,296 residents with portable ultrasound machines, and interviewed community members about daily habits, dog care practices, and food hygiene to understand infection risks. We found that CE was present in 0.34% of the screened population. Strikingly, more than 80% of all detected cases were in children and adolescents, suggesting recent infections and showing that transmission is still active. Most of the cysts detected (93%), were in active stages. Through a questionnaire completed by more than 2,200 villagers, we learned that people were at much higher risk of infection if they owned dogs (3 times higher risk), did not deworm their dogs (11 times higher risk), or fed dogs raw organs from livestock (3 times higher risk). Eating unwashed vegetables and fruits also increased the risk of infection, especially among women. By combining ultrasound and questionnaire findings, our work shows where prevention efforts should focus. Improving dog deworming, managing slaughter waste safely, and encouraging simple hygiene practices could prevent cases and reduce the burden of this disease in endemic communities of Kazakhstan.

## Introduction

The echinococcosis disease group, caused by various species of small tapeworms from the genus *Echinococcus*, is among the most common zoonotic diseases affecting humans. *Echinococcus* species are cestode parasites belonging to the family Taeniidae. The definitive hosts are carnivores, primarily members of the dog family (Canidae), while the intermediate hosts are typically herbivores (Perissodactyla and Artiodactyla). Humans serve as dead-end intermediate hosts, primarily affected by cystic echinococcosis (CE) and alveolar echinococcosis (AE), caused by *E. granulosus sensu lato* (*s.l*.) and *E. multilocularis*, respectively [1–4]. CE is the most prevalent form in both animals and humans, while AE is a more severe clinical condition in humans.

*E. granulosus s.l.* comprises a complex of species and genotypes, including *E. granulosus sensu stricto* (*s.s*.) (G1 and G3 genotypes), *E. equinus* (G4), *E. ortleppi* (G5), *E. canadensis* (G6/G7 cluster, G8 and G10), and *E. felidis* [1,2,4]. Each species may have specific transmission dynamics and life cycles linked to specific intermediate and definitive hosts, offering valuable insights into parasite biology and host range [5,6].

The adult worm inhabits the small intestine of dogs and other canids, which serve as definitive hosts. Eggs are released from gravid segments of the tapeworm and excreted in dog feces, contaminating soil, water, and vegetables [7,8]. Humans become infected by ingesting these eggs, leading to the development of echinococcal (or hydatid) cysts, space-occupying lesions primarily found in the liver (70%), lungs (19%) and other sites (11%) [7,9].

Understanding the life cycle of *Echinococcus* spp. is important for determining key risk factors for human infection. *E. granulosus s.s.,* commonly referred to as the sheep strain, has a domestic life cycle involving dogs and sheep, along with other livestock species [8,10]. It is well known that owning dogs and feeding them raw livestock offal significantly increases the human infection risk [11,12]. Since humans are dead-end hosts, no further transmission of the parasite occurs after humans become infected; however, CE can cause long-term disability, posing serious social and economic challenges in endemic regions [1,13].

CE is globally distributed, particularly in South America, North Africa, Asia, and Europe. Central Asian and many other countries are of particular interest due to the traditional practice of livestock breeding and the close contact between people and domestic animals such as dogs and sheep, which are the main hosts of the parasite [2,7,14,15]. Kazakhstan is among the countries most affected by echinococcosis [14,15]. Previous studies indicated that the southern regions (Turkestan, Zhambyl, Almaty and Zhetysu) of Kazakhstan are highly endemic for *E. granulosus s.l.*, with age-standardized incidence rates in Turkestan (formerly South Kazakhstan) at 9.93 per 100,000, Almaty at 7.65 per 100,000, and Zhambyl regions at 8.00 per 100,000 [15–17]. In recent years, due to urbanization and migration from rural areas, an increased number of CE cases have been consistently reported in southern cities such as Almaty and Shymkent. Published data indicated a CE incidence of 4.74 cases per 100,000 population in 2016 - similar to the 5.6 cases per 100,000 population in 2007 [6,15,16]. However, further large-scale studies are required to better understand the epidemiology of CE in this region.

The purpose of this study was to investigate the prevalence of CE in remote settlements of endemic regions in Kazakhstan and to identify the main risk factors contributing to the high incidence of this neglected parasitic disease.

## Materials and methods

### Ethics statement

The study protocol, which included a standardized questionnaire, informed consent forms, and information sheets in Kazakh and Russian, was approved by the Local Bioethics Commission of the Higher School of Medicine, Faculty of Medicine and Healthcare of Al-Farabi Kazakh National University (Institutional Review Board No. IRBA-516, dated November 10, 2022).

### Study design, duration, and ethical aspects

This was a cross-sectional, population-based ultrasound (US) screening study for CE, conducted between September 2023 and June 2024 by the Higher School of Medicine, Faculty of Medicine and Healthcare, Al-Farabi Kazakh National University, in collaboration with the A.N. Syzganov National Scientific Center of Surgery in Kazakhstan.

The clinical-epidemiological study was conducted in accordance with the Code of the Republic of Kazakhstan dated July 07, 2020 No. 360-VI ZRK, “On Public Health and the Healthcare System”.

A questionnaire survey was conducted to identify potential risk factors for human CE infection. Participants completed the standardized questionnaire prior to undergoing US screening. Information in the questionnaires included demographic data (age, gender, place of residence, and duration of residence in an endemic area), dog ownership and related practices (ownership within the past 5 years, even if no dog is currently owned; frequency of deworming; feeding dogs offal; cleaning dog habitats such as kennel or yard areas; proper disposal of dog feces), and the level of knowledge and practical habits related to personal hygiene (touching and/or petting dogs; eating raw, unwashed vegetables and/or fruits; nail-biting; neglecting hand washing before meals; using unfiltered water from open surface reservoirs (including natural or artificial sources unsuitable for drinking).

### Study area

**Fig 1:**
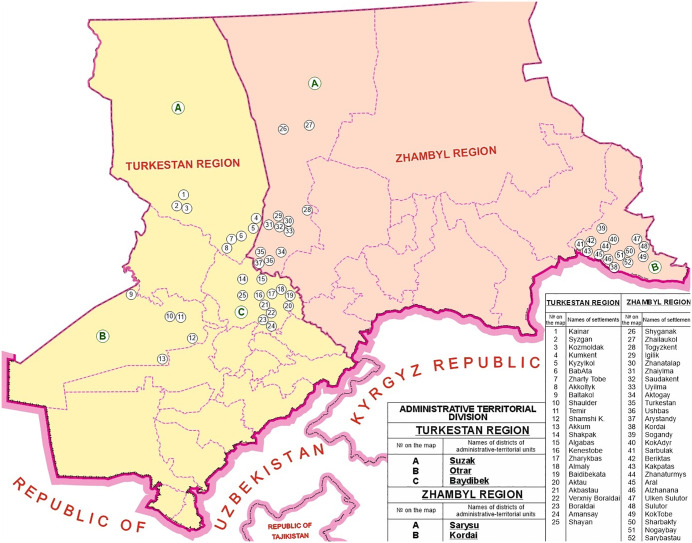
Ultrasound screening coverage of cystic echinococcosis-endemic villages in Turkestan and Zhambyl regions.

The study included residents of livestock-breeding communities in 51 pre-stratified remote villages across five districts in two endemic regions of Kazakhstan: 1) Zhambyl region, including Kordai and Sarysu districts; 2) Turkestan (formerly South Kazakhstan) region, including Baydibek, Otrar, and Suzak districts (Fig 1). Village stratification was carried out jointly with the responsible personnel of each Central District Hospital to select remote villages within the districts under their jurisdiction. Selected villages had populations of fewer than 2,000 residents, reported CE cases, lacked prior US screening in the past 5 years or more, and had limited medical infrastructure (i.e., no US equipment in local outpatient clinics).

### Population sampling

Prior to fieldwork, preparatory coordination was conducted with the administrations of the central district hospitals to stratify rural medical offices based on population size, distance from the district hospital, community lifestyle, and CE incidence. Medical personnel of rural outpatient clinics were informed in advance through information sheets and tasked with notifying residents about the upcoming screening via SMS, phone calls, or personal outreach.

Using a convenience sampling method, a total of 8,296 individuals voluntarily underwent abdominal US screening. Among them, 2,274 participants completed the standardized questionnaire. Eligible participants included residents of the target villages aged 5 years to > 60 years, with no gender restrictions. The questionnaire component was offered to participants aged 10 years to > 60 years. Previously diagnosed patients with CE were additionally eligible for screening, including those with known extrahepatic CE, those at stages CE4 and CE5, and those with a history of liver surgery within the past 3 years. All participants were required to have lived in the area for at least 5 years.

### Ultrasound examination

In this study, the Chison SonoBook6 Portable Ultrasound Diagnostic System (China, 2022) equipped with a C-3E, 2.0-6.8 MHz convex probe and a black-and-white printer, was used to screen the participants*.* Ultrasound examinations were performed by a qualified radiologist and three radiology residents (first- and second-year trainees). Screenings were conducted in medical offices in primary and secondary schools for school-aged children, as well as in mobile clinics*.*

Any detected cystic liver lesion, regardless of size, was independently re-examined by at least three sonographers from the screening team to confirm the diagnosis and determine subsequent steps for further diagnostic evaluation and treatment. Each patient received a detailed written sonographic report, including images and a summary of findings. Cyst staging was performed in accordance with the clinical protocol of the Ministry of Health of the Republic of Kazakhstan, based on the WHO-IWGE recommendations [18].

For all patients with cystic liver lesions superior or equal to 1 cm detected by US, 5 ml of blood was drawn from the cubital vein (with informed consent) for IgG ELISA testing for *Echinococcus*.

Patients with positive US findings were referred to the central hospital of their respective district for additional abdominal organ sonography using a stationary US machine, contrast-enhanced computed tomography scans, and chest X-ray. Treatment plans were determined based on these findings.

Following the US staging of echinococcal cysts and serological test, each patient with cystic liver involvement was registered with a diagnosis of CE by the local epidemiologist at the Central District Hospital. Patient management was subsequently guided by the WHO-IWGE recommendations. The positive patients were allocated to treatment and the results of the case managements were reported in this study. Family members of confirmed patients were invited to undergo abdominal US screening.

### Collection of CE data

Data on newly identified cases of СE were obtained from the Scientific and Practical Centre for Sanitary and Epidemiological Expertise and Monitoring (SPCSEEM) for the years 2023 and 2024. SPCSEEM serves as the lead center for infectious disease surveillance and monitoring of the sanitary and epidemiological situation in Kazakhstan. All new surgical CE cases, confirmed by histological examination of the chitinous layer using hematoxylin and eosin staining, were mandatorily registered with SPCSEEM. Surgeons were required to submit biological samples (chitinous layer) to the hospital or clinic pathology departments. Pathologists notified the hospital or clinic epidemiological surveillance office of each confirmed case. Hospital administrators subsequently reported these cases to the regional public health centers. An emergency epidemiological alert was issued to the local (regional/district/city) Department of Sanitary and Epidemiological Control at the patient’s place of residence. Case information was transmitted monthly to SPCSEEM’s central data processing center. The collected data includes demographic, clinical and laboratory information, as well as the length of hospital stay. The SPCSEEM infectious disease registry records only surgical cases of CE. Relapse cases and early-stage CE cases with small cysts managed through conservative therapy are not included in the registry.

### Questionnaire data collection

Ultrasound surveys were complemented by both qualitative and quantitative data collection using a standardized questionnaire containing closed- and open-ended questions. Screening for CE began with an introductory meeting at the screening site. Participants received an oral explanation detailing the purpose of the screening, the diagnostic procedures involved, the importance of providing accurate responses, the risks CE poses to humans and animals, its routes of transmission, and recommended preventive measures.

### Risk factor analysis

To identify potential risk factors, the questionnaire collected information on common behavioral and lifestyle characteristics, including personal hygiene (e.g., hand washing before meals), food consumption habits (eating unwashed or raw produce), water sources (drinking from open water sources), living environment (rural or urban, access to medical care, socioeconomic status), pet ownership, farming activities, availability of veterinary care, occupation (office work, agriculture, livestock farming), and education level (none, primary/secondary, college, university). Risk factors were analyzed into two categories: behavioral factors, modifiable through individual actions, habits, or preventive measures, and socioeconomic factors, related to living conditions and environment.

### Statistical analysis

Data were summarized using absolute frequencies and percentages. Univariate analysis was performed using the chi-square (χ²) test with likelihood adjustment. Variables showing a statistically significant association with CE presence were included in a multivariate logistic regression model using the forced entry method. Odds ratios (OR) along with 95% confidence intervals (CI) were calculated to estimate the strength of association between risk factors and CE infection.

Age- and gender-specific incidence rates (per 100,000 population) as well as age-standardized incidence rates, were calculated for CE surgical cases reported from regions of Kazakhstan, including the cities of Astana, Almaty, and Shymkent, from 2022 to 2024.

A p-value < 0.05 was considered statistically significant. All analyses were performed using IBM SPSS Statistics software (version 19). Only risk factors that significantly increased the probability of CE infection were included in the multivariate model.

## Results

### Incidence of CE in Kazakhstan

According to data from SPCSEEM, the overall incidence of CE in Kazakhstan significantly decreased in 2024 (n = 641; 3.20 per 100,000 population) compared to 2022 (n = 792; 4.8 per 100,000 cases; p < 0.0001) and 2023 (n = 748; 3.77 per 100,000 cases; p = 0.0018). This rate is below the incidence threshold established by the WHO for hyperendemic area for echinococcosis, which is 5.0 cases per 100,000 inhabitants. However, CE incidence remains high in several endemic regions, including Turkestan, Zhetysu, Almaty, West Kazakhstan, and Zhambyl (Table 1).

**Table 1:** Surgical incidence of cystic echinococcosis per 100,000 populations by region and major cities, 2022–2023 (SPCSEEM data).

	Absolute numbers (incidence/100,000)			Incidence difference, %	
Regions	2022	2023	2024	2022-2023	2023-2024
Abay	9 (1.41)	9 (1.48)	14 (2.30)	+0.07	+0.82
Akmola	19 (2.49)	11(1.40)	9 (1.14)	-1.09	-0.26
Aktobe	49 (5.38)	34 (3.65)	35 (3.73)	-1.73	+0.08
Almaty	**121** (**8.05**)	**91** (**6.27**)	**86 (5.67)**	-1.78*	-0.65
Atyrau	6 (0.90)	11 (1.59)	7 (0.99)	+0.69	-0.60
East Kazakhstan	13 (1.81)	15 (2.05)	10 (1.38)	+0.24	-0.67
Jambyl	**69** (**6.01**)	**65** (**5.34**)	**64 (5.23)**	-0.67	-0.11
Zhetysu	**29** (**4.29**)	**69** (**10.23**)	**62 (8.88)**	+5.94*	-1.35
West-Kazakhstan	**61** (**9.27**)	**43** (**6.26**)	**40 (5.77)**	-2.91	-0.49
Karaganda	26 (2.15)	23 (1.93)	16 (1.41)	-0.21	-0.52
Kostanay	5 (0.58)	7 (0.81)	2 (0.24)	+0.23	-0.57
Kyzylorda	25 (2.94)	25 (3.90)	20 (2.38)	+0.96	-1.52
Mangistau	20 (2.64)	29 (3.73)	22 (2.80)	+1.09	-0.93
Pavlodar	6 (0.76)	5 (0.65)	3 (0.40)	-0.11	-0.25
North-Kazakhstan	8 (1.47)	6 (1.12)	5 (0.94)	-0.35	-0.18
Turkestan	**227** (**11.21**)	**212** (**10.34**)	**161 (7.52)**	-0.87	-2.82*
Ulytau	4 (1.84)	4 (1.86)	6 (2.71)	+0.02	+0.85
Cities					
Astana city	1 (0.08)	1 (0.07)	1 (0.07)	-0.01	0.00
Almaty city	27 (1.26)	29 (1.30)	19 (0.85)	+0.04	-0.45
Shymkent city	67 (6.15)	47 (4.03)	59 (4.83)	-2.12*	+0.80
Kazakhstan (total)	**792** (**4.8**)	**748** (**3.77**)	**64 1(3.20)**	-0.31*	-0.57*

* Statistically significant difference P ≤ 0.05.

Despite the national decline, statistically significant reductions in CE incidence were recorded in specific areas, including Almaty (p = 0.028) and Zhetysu (p < 0.0001), Shymkent city (p = 0.045), and Turkestan region (p = 0.0061).

**Fig 2:**
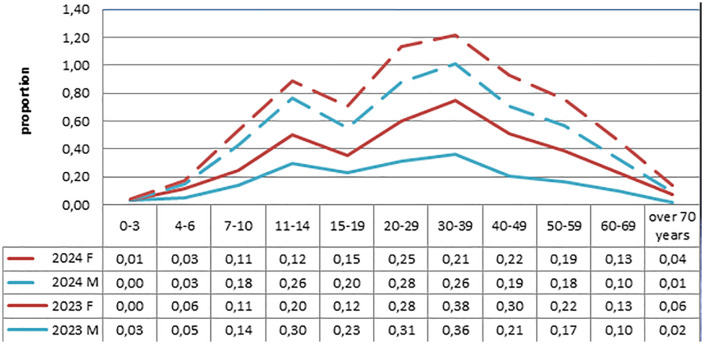
Comparison of age and gender distribution of surgical cases of cystic echinococcosis in 2023 and 2024 (SPCSEEM data).

In 2023, surgical CE cases were slightly higher in men (n = 379; 1.91 per 100,000) than in women (n = 369; 1.87 per 100,000), with no statistically significant difference (p = 0.3086). In 2024, the incidence was significantly higher in men (n = 344; 1.71 per 100,000) than in women (n = 297; 1.48 per 100,000; p = 0.0144). In 2023, 245 cases (32.7%) occurred among individuals aged 0–19 years, compared to 222 cases (34.6%) in 2024. Among adults, the highest incidence was observed in the 30–39 age-group, while among children, the peak incidence occurred in the 11–14 age-group, based on SPCSEEM’s age classifications (Fig 2).

### Demographic characteristics of ultrasound screening participants

A total of 8,296 residents voluntarily participated in US screenings, representing 0.247% of the total population in Zhambyl and Turkestan regions, and 2.264% of the population within the five selected endemic districts (as of October 2023). Participation by district was as follows: Kordai - n = 2,505; 1.579% of 158,595; Sarysu - n = 1,952; 4.421% of 44,154; Baidibek - n = 1,669; 3.414% of 48,877; Otrar - n = 482; 0.934% of 51,571; and Sozak - n = 1,688; 2.668% of 63,258. Significantly more children aged 0–18 years (n = 6,273; 75.6%) than adults (n = 2,023; 24.4%) participated (χ² = 1722.3, 95%CI [48.9; 53.3], p < 0.0001).

Among all US survey participants, 2,274 individuals (29.4%) completed the questionnaire, with 1,222 (53.7%) from the Zhambyl region and 1,052 (46.3%) from the Turkestan region.

All adult participants (n = 2,023) underwent both US examination and **questionnaire survey**: Kordai - n = 552; 27.3%, Sarysu - n = 363; 17.9%, Baidibek - n = 651; 32.2%, Otrar - n = 204; 10.1%, and Sozak - n = 253; 12.5%. Among children aged 0–18 years (n = 6,273), only 555 (8.8%) completed the questionnaire survey: Kordai - n = 2,108 with 155 (2.5%), Sarysu - n = 1,715 with 126 (2.0%), Baidibek - n = 1,029 with 11 (0.2%), Otrar - n = 527 with 249 (3.9%), and Sozak - n = 1,449 with 14 (0.2%) of children.

The age of participants ranged from 7 to 84 years, with a mean age of 38.06 ± 20.6 years. Women were significantly more represented (n = 1,592; 70%) than men (n = 682; 30%), (χ² = 313.5, 95%CI [35.8; 44.0], p < 0.0001). The female-to-male ratio was 0.9:1 and among children (0–18 years) and adults it was 3.3:1.

**Fig 3:**
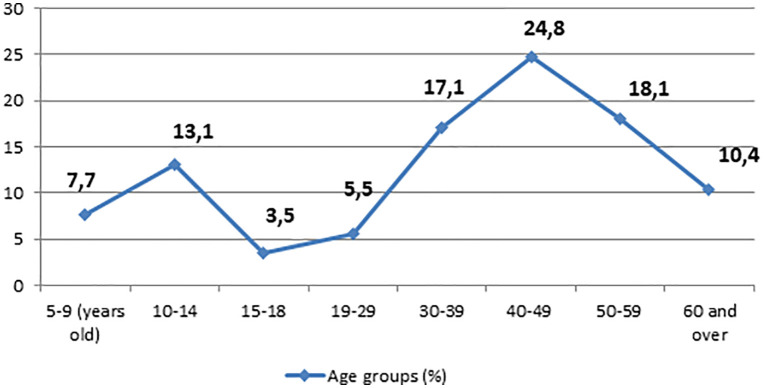
Age characteristic of participants in ultrasound screening and questionnaire survey for cystic echinococcosis.

The mean age of children and adults who completed the questionnaire was 12.84 ± 1.98 years, while the mean age of adult respondents was 46.34 ± 11.90 years (Fig 3).

Among the school-based US survey participants, the majority were school children (n = 510; 22.4%) and preschool children (n = 45; 2.0%). Among adults, the largest group consisted of seasonal (temporary) agricultural workers, including those employed on farms and peasant holdings (n = 1,131; 49.7%). Smaller proportions included employees (n = 321; 14.1%), pensioners (n = 233; 10.2%), and general workers (n = 34; 1.5%).

### Ultrasound examination findings

Ultrasound screening of 8,296 rural residents identified 28 individuals (0.34%) with CE. Most detected cysts were active lesions, with 26 cases (92.85%) classified as CE1, CE3a and CE3b. Only two cases (7.14%) showed features of inactive cysts (CE5), and no CE4-stage degenerative cysts were observed. In addition to confirmed cases, US revealed five CL-stage cysts (0.06%), representing simple unilocular lesions lacking diagnostic characteristics of echinococcosis but requiring follow-up. No cases of AE were identified during this screening.

A case of familial CE was identified among family members who were invited to undergo US examinations. One of the two newly identified cases of CE in the village of Beriktas, Kordai district, was an 11-year-old sister (CE1) of a 16-year-old (CE3a) and a 13-year-old (CE3b).

The distribution of newly identified CE cases varied across districts. Baidibek reported four cases (0.24%), Otrar three cases (0.62%), Sozak six cases (0.36%), Kordai eight cases (0.32%), and Sarysu ten cases (0.51%). These patterns corresponded to recent surgical case notifications, which in October 2023 totaled 15 cases in the Zhambyl region and 13 cases in the Turkestan region. Statistical comparison showed no significant difference in CE risk between the two regions (OR = 0.995, p = 0.987), suggesting a similar level of ongoing transmission across the study areas.

The proportion of newly identified CE cases relative to the number of screened residents was as follows: Baidibek: n = 4; 0.24% of 1,669; Otrar: n = 3; 0.62% of 482; Sozak: n = 6; 0.36% of 1,688; Kordai: n = 8; 0.32% of 1,952, and Sarysu: n = 10 cases; 0.51% of 1,952.

To assess long-term recurrence, 16 previously treated participants (≥ 5 years post-treatment) with postoperative residual cavities (RC) were examined. No recurrences were detected, supporting that stable RC in this population may not represent active infection. Table 2 presents the distribution of CE cases by age group, WHO-IWGE cyst stage, and treatment outcomes.

**Table 2:** Distribution of Cyst stages (WHO-IWGE classification) by age group and cysts size.

	CE1	CE2	CE3a	CE3b	CE5	Total	Recurrence	Regression	Residual cavity
**CE (%)**	17 (60.7%)	3 (10.7%)	2 (7.1%)	4 (14.3%)	2 (7.1%)	28	1	6 (21.4%)	16 (0.19%)
**Children (%)**	14 (50.0%)	2 (7.1%)	2 (7.1%)	4 (14.3%)	1 (3.6%)	23 (82.1%)	–	3 (10.7%)	–
1-4 years	–	–	–	–	–	–	–	–	–
5-8 years	1 (3.6%)	–	–	–	–	1 (3.6%)		1 (3.6%)	
9-14 years	10 (35.7%)	1 (3.6%)	–	3 (10.7%)	1 (3.6%)	15 (53.6%)	1 (3.6%)	2 (7.1%)	–
15-18years	3 (10.7%)	1 (3.6%)	2 (7.1%)	1 (3.6%)	–	7 (25.0%)	–	–	–
**Adults (%)**	3 (10.7%)	1 (3.6%)	–	–	1 (3.6%)	5 (17.9%)	–	3 (10.7%)	16 (0.19%)
19-29 years	–	1 (3.6%)	–	–	–	1 (3.6%)	–	1 (3.6%)	0
30-39years	1 (3.6%)	–	–	–	–	1 (3.6%)	–	–	1 (6.2%)
40-49 years	1 (3.6%)	–	–	–	1 (3.6%)	2 (7.1%)	–	1 (3.6%)	10 (62.5%)
50-59 years	1 (3.6%)	–	–	–	–	1 (3.6%)	–	1 (3.6%)	0
> 60 year	–	–	–	–	–	–	–	–	5 (31.3%)
**Average** **Cystic** **size (cm)**	4.9 ± 2.8	9.3 ± 4.5	6.9 ± 0.6	8.1 ± 1.6	3.6 ± 2.0	5.9 ± 3.2	5.3	3.4 ± 0.9	

Age-specific analysis revealed a marked concentration of infections among younger participants. Ultrasound screening did not detect new cases of CE among individuals aged 0–8, or ≥ 60 years, but the overall burden was highest in adolescents and young adults. No infections were identified among individuals aged ≥ 60 years, indicating either reduced exposure or a lower likelihood of detecting early-stage cysts in this age group. The predominance of CE1-stage lesions, particularly in younger participants, suggests recent and active transmission within these communities.

Ultrasound follow-up also provided information on treatment outcomes. Among the detected cases, six individuals with CE1 cysts demonstrated progression to inactive stages (CE4/CE5) after receiving two to three courses of albendazole, administered according to body weight. One patient experienced recurrence following PAIR combined with albendazole therapy. A CE1 lesion reappeared at one year, prompting a six-month retreatment course; subsequent imaging showed evolution to CE4 with a positive serologic response. Albendazole was discontinued, and further US and serologic monitoring were recommended (Table 3).

**Table 3:** Treatment of Cystic echinococcosis according to stages.

	CE1	CE2	CE3a	CE3b	CE5	Total	Recurrence	Regression	Residual cavity
**Treatment (%)**	17 (60.7%)	3 (10.7%)	2 (7.1%)	4 (14.3%)	–	26 (92.9%)	–	–	–
ABZ/ Surgical/ ABZ	8 (28.6%)	2 (7.1%)	2 (7.1%)	4 (14.3%)	–	16 (57.1%)	1 (3.6%)	15 (53.6%)	–
ABZ	9 (32.1%)	1 (3.6%)	–	–	–	10 (35.7%)	–	5 (17.9%)	–
**Observation (%)**	–	–	–	–	2 (7.1%)	2 (7.1%)	–	–	16 (0.19%)

Overall, the US findings highlighted ongoing community-level transmission, a high proportion of early-stage cysts, and age patterns consistent with recent exposure, underscoring the importance of US as a sensitive tool for detecting early infections in endemic rural settings.

### Risk factor questionnaire

Analysis of age distribution demonstrated that adolescents presented the highest prevalence of CE. The risk of developing CE among individuals aged 9–18 years was significantly elevated (OR = 14.82, z-statistic = 5.44, 95%CI [5.6–39.2], p < 0.0001). The influence of risk factors on the incidence of CE is presented in Table 4.

**Table 4:** Univariate and multivariate analysis of risk factors for cystic echinococcosis.

	n	CE+	CE-	OR	95%CI	P value
**Univariate analysis**						
Gender						
Men	682	12	670	1.76 ^γ^	[0.8;3.7]	0.140
Women	1,592	16	1,576			
Owning yard dog for the last 5 years?						
Yes	1,182	22	1,160	3.17^γ^	[1.3;7.8]	0.012*
No	1008	6	1,002			
Deworming dog for the past 3–5 years						
No	1,605	27	1,578	11.12^γ^	[1.5;82.0]	0.018*
Yes	651	1	650			
Keeping the dog on a leash?						
No	1,382	11	1,371	0.41 ^β^	[0.2;0.9]	0.023*
Yes	892	17	875			
Feeding dogs with raw offal?						
Yes	461	8	453	1.58^γ^	[0.7;3.6]	0.275
No	1,813	20	1,793			
Cleaning up after dogs?						
Yes	494	2	492	0.27^β^	[0.07;1.2]	0.078
No	1,780	26	1,754			
Eating unwashed vegetables and fruits?						
Yes	144	6	138	4.16^γ^	[1.7;10.4]	0.002*
No	2,129	22	2,107			
The habit of taking wild grass or a straw from the ground into your mouth?						
Yes	86	3	83	3.13^γ^	[0.9;1.1]	0.066
No	2,187	25	2,162			
Work at a meat processing plant?						
Yes	71	0	71	0.53 ^β^	[0.03;8.8]	0.661
No	2,203	28	2,175			
**Multivariate analysis**						
No deworming of dog for the last 3–5 years and (Yes/No) feeding dogs with raw offal						
Yes	171	7	164	3.06^γ^	[1.3;7.3]	0.012*
No	1,452	20	1,432			
Yes deworming of dog for the last 3–5 years and (No/Yes) keep the dog on a leash						
No	166	0	166	1.0 ^α^	[0.03;23.9]	0.985
Yes	485	1	484			
Gender distribution (female) and (Yes/No) Eating unwashed vegetables and fruits						
Yes	98	4	94	5.25^γ^	[1.7;16.6]	0.005*
No	1,493	12	1,481			
Gender distribution (male) and (Yes/No) Eating unwashed vegetables and fruits						
Yes	46	2	44	2.85 ^α^	[0.6;13.4]	0.186
No	636	10	626			
Gender distribution (female) and (Yes/No) The habit of taking wild grass or a straw from the ground into your mouth						
Yes	52	2	50	4.35 ^γ^	[0.9;19.7]	0.05*
No	1,539	14	1,525			
Gender distribution (male) and (Yes/No) The habit of taking wild grass or a straw from the ground into your mouth						
Yes	34	1	33	1.75 ^γ^	[0.2;14.0]	0.596
No	648	11	637			

*Statistically significant difference P ≤ 0.05.

OR: Odds ratio; α: OR = 1 means that the odds are equal in both groups; β: OR < 1 means that the event has an inverse relationship and chance to occur in the second group; γ: OR > 1 means that the event is directly related and has a chance of occurring in the first group.

**Fig 4:**
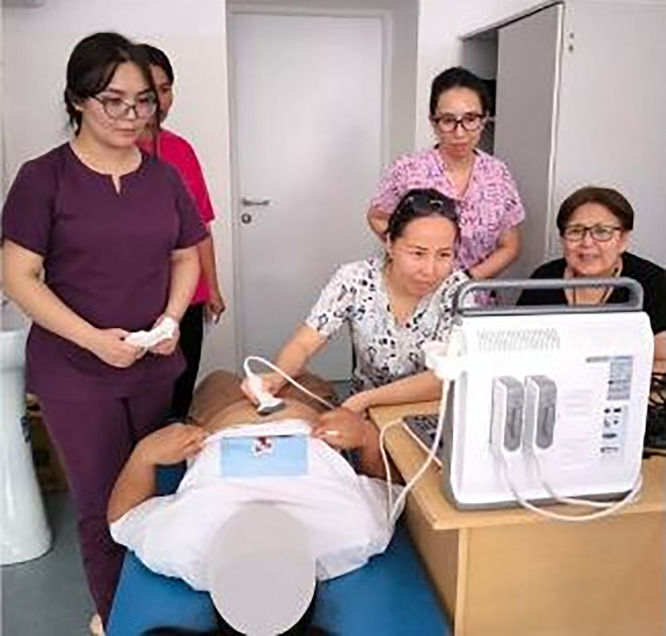
Ultrasound screening coverage of cystic echinococcosis. Endemic Zhanaturmys village, Kordai district, Zhambyl region.

**Fig 5:**
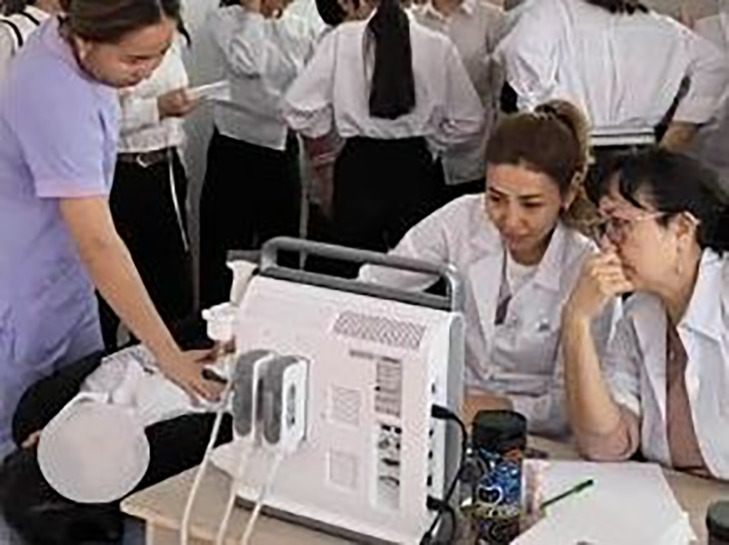
Ultrasound screening coverage of cystic echinococcosis. Endemic Boraldai village, Baydibek district, Turkestan region.

High-risk groups included children and adolescents, particularly those exposed to yard dogs or domestic cats, and those who consumed unwashed vegetables and fruits. A lack of regular deworming of dogs (i.e., fewer than two treatments per year over the past 3–5 years) was significantly associated with an increased risk of CE infection. Multivariate analysis confirmed that the absence of dog deworming and feeding dogs raw offal were the factors most strongly associated with CE infection. Among women, higher risk was observed in those who reported consuming unwashed vegetables or fruits and engaging in behaviors such as placing grass or other objects from the ground into their mouths. (Figs 4 and 5). The interaction of risk factors has a synergistic impact on increasing the risk of CE.

The attributable risk of CE due to the absence of regular dog deworming was 90.87%, while the population attributable risk was 87.62%.

Pearson correlation analysis revealed a weak positive association between feeding dogs raw offal and CE incidence (r = 0.430, p = 0.003).

## Discussion

This study investigated the present status of CE in endemic regions of Kazakhstan through US screenings and community-based questionnaires. The aim was to identify key transmission factors of *E. granulosus* to humans. CE is recognized as a rare orphan disease in most European countries, with southeastern Europe as the epicenter [3]. However, a decline in incidence has been observed in many Mediterranean countries attributed to improved hygiene and effective control programs [3,6,19]. In contrast, in Central Asia, including Kazakhstan, control measures remain largely ineffective [6,20]. In Kyrgyzstan, both AE and CE have reached epidemic proportions in recent years [21,22]. A high incidence of CE additionally persists in Uzbekistan and Tajikistan [14,20]. According to literature [23] and WHO recommendations, deworming dogs four times annually is more effective. However, Kazakhstan’s national guidelines, as implemented by SPCSEEM, currently recommend only twice annually, which may contribute to sustained transmission in endemic areas. A higher frequency of AE has been noted in regions of Kazakhstan bordering China (e.g., Almaty, Zhetysu, and Abay), as well as in North Kazakhstan, Pavlodar, and Kostanay (bordering Siberia), and the East Kazakhstan region (bordering Russia’s Altai region).

From 2022 to 2024, the number of registered surgical CE cases in Kazakhstan demonstrated a statistically significant decline (p < 0.0001). Nonetheless, incidence rates remain high in Almaty, Zhambyl, Zhetysu, West Kazakhstan, and Turkestan, where livestock breeding is a primary occupation. According to the *Agency for Strategic Planning and Reforms of the Republic of Kazakhstan, Bureau Of National Statistics* (https://stat.gov.kz/en/) these regions have demonstrated consistent growth in livestock numbers across private, peasant, and farm households between 2023 and 2024.

In the Zhetysu region, the observed rise in CE incidence in 2023 by 5.94% is likely due to incomplete reporting for 2022, which covered only the final five months following the administrative division of the former Almaty region into two separate parts.

The overall reduction in CE cases may additionally be associated with increased urbanization, as rural residents migrate to cities due to unemployment in smaller settlements. Analysis of CE surgical cases by age and sex distribution of surgical cases of CE, according to SPCSEEM data for 2023–2024, showed that the 30–39 year age group presented the highest susceptibility, followed by the 20–29 age group and children aged 11–14 years. These findings are consistent with previous studies [15,20]. However, gender distribution within the 30–39 age group did not demonstrate a strong female predominance: in 2023, rates were 0.38 (f) and 0.36 (m), while in 2024 they were 0.21 (f) and 0.26 (m), contrasting earlier findings [15]. This discrepancy is likely due to the fact that our analysis of morbidity dynamics over the years showed variability in indicators among men and women, whereas the previous study performed a summary analysis of morbidity in men and women over the past few years. Notably, the 20–29 and 30–39-year age groups represent the economically active population, often engaged in agricultural labor or self-employed in rural and peri-urban settings. Their involvement in farming, herding, and household chores increases exposure to the parasite.

This cross-sectional study was conducted in alignment with the roadmap for Neglected Tropical Diseases 2021–2030 [24], which calls for intensiﬁed control of CE in endemic countries. The work was supported by the Ministry of Science and Higher Education of Kazakhstan and focused on 51 remote villages in five districts across two endemic regions.

This screening study included primarily children (77.6%), as this group is directly linked to educational institutions. The adult population accounted for 22.4%of participants, most of whom were in the 40–49 age group and predominantly women (70.01%). Adults were mainly seasonal workers (peasants) or involved in farming households (49.7%). The relatively low participation of adults, especially men, may be attributed to the migration of the working-age population to urban centers due to high unemployment in remote villages.

The US detected prevalence of CE among the rural population was 0.34%, with a higher prevalence in younger age groups. The infection was significantly associated with the presence of yard dogs and inadequate or absent dog deworming. All identified CE cases were newly diagnosed. Of these, 92.85% were active cysts (WHO stages CE1–CE3b), predominantly found in young individuals, while inactive cysts (CE5) accounted for 7.14%, distributed equally between adults and youths.

Previous studies have demonstrated that rural populations are 1.83 to 2.26 times more likely to contract CE, with villages of low socioeconomic status presenting a 1.68 times higher risk [12,25]. In our study, the observed rate of 60.7% of newly identified cases of CE1 cysts, with half occurring in children and adolescents, and 10.7% in adults, suggests a recent or ongoing transmission. While previous studies have indicated a higher cumulative risk of CE in older individuals due to prolonged exposure [12,26,27], adolescents remains a key at-risk group [28,29]. These findings support the hypothesis that initial infection may occur in early school-age children, highlighting the need for targeted health education and prevention programs starting at a young age.

The findings showed that 21.43% of the CE cases occurred in individuals who reported no dog ownership over the past 5 years. As reported in other studies, this likely reflects a possible environmental contamination from feces of infected stray dogs [30,31]. Field observations during the screening revealed that stray dogs frequently congregate in schoolyards, particularly near playgrounds where children gather during breaks and after classes, and near grocery stores often visited by children. Such dogs are a major source of infection for both rural and urban residents through contaminated feces [30,32]. Prior studies have found that dog ownership increases the risk of infection by 3.54 times [12].

New CE cases were predominantly detected in underdeveloped villages with poorly organized veterinary services and insufficient dog deworming. The wide spread practice of feeding dogs raw offal was associated with an attributable risk of 36.43%. Our findings showed that regular deworming of dogs (at least twice a year) could potentially prevent up to 87.6% of CE cases in endemic areas. While earlier studies down played the significance of irregular deworming [12,25], our data demonstrated that the absence of dog deworming over the past 3–5 years increases the risk of human CE infection by 11.12 times. In villages, where residents confirmed the regular deworming of dogs at least twice a year by the local veterinary services, no new cases of CE were identified during the screening period.

Among some respondents who reported deworming their dogs in the past 3–5 years but continued to feed them raw offal, the likelihood of developing CE increased by 3.06 times. However, multivariate analysis showed that, despite dogs being dewormed twice within this period, factors such as whether the dog was kept on a leash or whether the owner cleaned up after the dog did not significantly influence the risk of developing CE.

Eating unwashed vegetables and fruits increased the odds of CE infection five times among women and 2.85 times among men. Additionally, the habit of putting wild grass or straw from the ground into the mouth increased the risk of CE infection by four times among women and 1.7 times among men. Women are often involved in agricultural work, often growing and harvesting fruits and vegetables, as well as preparing food, feeding dogs, making them more susceptible to infection. This supports the hypothesis that environmental contamination is a major risk factor for infection. All households in the villages have access to drinking water through individual or public water pipes. Despite this, many residents purchased bottled drinking water, although low-income families often cannot afford it and thus rely on tap water. In the villages, tap water comes directly from artesian wells and is pumped by a pumping station to a water tower, from where it flows by gravity to residents’ homes. The system is closed, preventing contamination of the tap water from outside. It should be noted that adequate sanitary monitoring of water towers and artesian wells in remote villages is not carried out. Tap water is commonly used for household purposes such as washing dishes, vegetables, fruits, and hands. Most homeowners purify and filter their tap water themselves using household filters. Outdated water towers often fail, and problems are not repaired in a timely manner. In economically underdeveloped remote villages, there are no public utilities, so the issue of providing residents with tap water can be delayed. This forces residents to use other water sources for household purposes, including open water bodies. Meanwhile, open reservoirs and small irrigation ponds, which serve as water sources for livestock, flocks and stray dogs. Children frequently swim in these bodies of water during summer, often without adult supervision, especially in the southern regions of Kazakhstan. Unfortunately, the cleanliness of these reservoirs is not adequately monitored and may contribute to increasing CE transmission.

Sociological surveys of rural populations in endemic areas [11,26] have identified several key factors contributing to the high incidence of CE in Kazakhstan: low level of public awareness about preventive measures, unrestricted access of dogs to raw offal, a lack of designated slaughter and waste disposal sites accessible to the community, and insufficient dog deworming efforts. However, during the screening, participants were given an oral explanation of the survey’s importance and the importance of truthful responses to questions about risk factors. This undoubtedly influenced their awareness of the СE problem and, in some cases, may have influenced their responses to the questionnaire.

The attributable risk of CE in this study suggests that most CE cases in the population may be attributable to the lack of regular dog deworming, highlighting it as a potential driver of infection.

The identified weak positive Pearson correlation suggests that communities where dogs are more often fed with raw offal tend to have slightly higher CE incidence. While the association is not strong, it still supports the role of unsafe offal disposal and dog feeding practices in maintaining transmission.

Due to the incubation period of CE, the precise routes of infection in Kazakhstan remain incompletely understood, warranting further research in rural endemic regions. Transmission of the parasite to humans likely occurs through consumption of contaminated food and water or via the alimentary transmission of parasite eggs from contaminated soil and surfaces to the mouth (“hand-to-mouth” route), which may be as important as direct contact with dogs [8,33]. Further studies are needed to elucidate transmission dynamics among humans and evaluate the role of water sources, wild animals, and domestic animals in endemic regions of Kazakhstan.

## Conclusions

The key risk factors for CE in endemic villages identified in this study include unrestricted access of domestic and stray dogs to raw, unprocessed meat by-products, home slaughtering of livestock, improper waste disposal, insufficient frequency of dog deworming, and low public awareness of preventive measures. These findings highlight the urgent need for coordinated efforts to develop and implement effective CE control and prevention strategies. Although the highest observed incidence of CE was observed among adolescents, our data suggest that younger children should be prioritized as a target group for health education and preventive interventions. The results of this study provide valuable evidence to guide targeted control measures and inform public health policies aimed at reducing the burden of CE in Kazakhstan.

### Limitations

The registration of new cases of AE and CE in Kazakhstan is managed through a single registry, SPCSEEM. However, this registry does not fully reflect the true incidence of echinococcosis in the country. It primarily includes surgical cases and excludes early-stage CE patients who undergo conservative medical treatment with albendazole. Additionally, cases with inactive cysts at stages CE4 and CE5, which are managed on an outpatient basis without requiring surgery, are not captured in the registry. In other words, SPCSEEM overlooks non-surgical and inactive cases of CE, leading to under reporting. To address these gaps, the Syzganov National Surgery Center and SPCSEEM have jointly developed an action plan to improve case registration. Another important limitation is age bias. Since CE remains a symptomatic for many years and infection risk increases with age, and the screening likely did not capture the full extent of the disease. This suggests that the actual incidence of CE in Kazakhstan is under estimated.

Several factors may have contributed to an underestimation of the actual incidence of CE or a bias in the findings in this region, namely:

- Age-bias associated with the migration of the working-age adults population to urban centers due to high unemployment in remote villages;
- School-age children underwent US screening; however, not all voluntarily participated in the accompanying questionnaire survey. This was partly due to the fact that screenings and surveys were conducted during school hours, prompting many children to return quickly to their classes after the US. This likely contributed to the lower number of the questionnaire survey participants compared to the number of US screening participants;
- Employment challenges in remote rural villages contribute to low adult participation in screenings. Many working-age adults were absent from their homes during screening periods, working in larger settlements or nearby districts, while their children remained under the care of a single parent or close relatives;
- Coinciding school holidays contributed to incomplete screening coverage of the child population in villages in the Otrar district.

## Supporting information

S1 Protocol The study protocol was approved by the local bioethics committee. (PDF)
