# Supplementary material for: Population-based ultrasound prevalence and risk factors for cystic echinococcosis in endemic Kazakhstan
Source: PLoS Negl Trop Dis. 2026 Mar 16;20(3):e0014126. doi: 10.1371/journal.pntd.0014126 (PMC13004503; doi:10.1371/journal.pntd.0014126)
Supplement: S1 Protocol — (PDF) [file pntd.0014126.s001.pdf]

<table>
<tr><th>№</th><th colspan="3">Study protocol</th></tr>
<tr><td>1</td><td>Project</td><td colspan="2">AP19676676 "Development of a molecular epidemiological map of the prevalence of echinococcosis in the human population of Kazakhstan"</td></tr>
<tr><td>2</td><td>Head of the Project</td><td colspan="2">Ismailova Gulziya Nurtazaevna, higher education, Candidate of Medical Sciences (PhD), Associate Professor</td></tr>
<tr><td></td><td>Priority direction</td><td colspan="2">6. Science of life and health</td></tr>
<tr><td>3</td><td>The Interstate rubricator</td><td colspan="2">76 - Medicine and health care: (76.29.62 - Radiology and medical radiology, 76.29.00 - Clinical medicine); 76.00.00 - Medicine and healthcare.</td></tr>
<tr><td>4</td><td>Type of research</td><td colspan="2">Applied</td></tr>
<tr><td></td><td>Grant funding (for the entire duration of the project and by years, in thousand tenge)</td><td colspan="2">Total - 92,466,323 million tenge, by years:
2023 – 27 443666 million tenge,
2024 – 32 992794 million tenge,
2025 – 32 029863 million tenge.</td></tr>
<tr><td>5</td><td>Start and end date of the project, its duration in months:</td><td colspan="2">February 2023 - December 2025, 35 months.</td></tr>
<tr><td rowspan="3">6</td><td rowspan="3">Design studies</td><td colspan="2">Prospective observational study</td></tr>
<tr><td>Main group - A</td><td>Control group - B</td></tr>
<tr><td colspan="2">Group A - single or multiple cystic changes in the liver detected by ultrasound;
Group B - ultrasound intact patients</td></tr>
<tr><td rowspan="2">7</td><td>Criteria for inclusion of patients in the study</td><td colspan="2">Men and women, ages 16 and over;
Child population aged 5 to 15 years.
Never had echinococcosis
Complaining of abdominal pain
Registered, diagnosed with inactive echinococcosis
Removed from dispensary observation, with a diagnosis of echinococcosis.</td></tr>
<tr><td>Criteria for exclusion of patients from the study</td><td colspan="2">Early age up to 5 years;
Vulnerable patients, pregnant women
Patients with an established diagnosis of echinococcosis, during the follow-up period (5 years).</td></tr>
<tr><td>8</td><td>Informed consent (Forms applied)</td><td colspan="2">Participants are given an oral explanation of the purpose of screening, the diagnostic procedures used, the importance of providing accurate results, the risks associated with CE to humans and animals, the routes of transmission and the recommended preventive measures.
Distribution of Booklet and Information Sheets</td></tr>
<tr><td>7</td><td>Methods</td><td>Group A</td><td>Group B</td></tr>
<tr><td>7.1</td><td>Stratification of 21 (3 each) settlements of endemic (Turkestan, Zhambyl, Almaty / Zhetysu) and central (Akmola, Karaganda and Kostanai) regions of Kazakhstan. With a population of about 2,000 people engaged in cattle breeding or</td><td>Solitary cysts of echinococcosis (indicating the lobe, segment of the liver);
Multiple cysts of echinococcosis (with the defeat of one lobe, with the defeat of both lobes).</td><td>No cystic changes</td></tr>
</table>

	having farm animals in a private yard.		
7.2	Stratification of population by method of questioning (Standardized questionnaire applied)	Sociological survey, by questioning the participants of the study with the collection of socio-demographic data (gender, age, address, level of education, profession) and information on risk factors for living in (endemic southern regions of Kazakhstan); clarification of the place of residence throughout life (endemic regions, rural areas); occupation during life; contact with dogs (private, hunting, yard, service and domestic); contact with farm animals; feeding dogs raw internal organs of animals; the use of unwashed berries (strawberries, strawberries, etc.); the habit of taking and biting into the mouth wild herbs, etc .; employees of livestock farms, meat processing enterprises, slaughterhouses, animal disposal and burial facilities and family members; cutting and sale of meat, internal organs; owning a dog within the last 5 years; work as a shepherd, hunter, huntsman and family members; work in a workshop for the processing of fur raw materials and family members; veterinarian and family members; employee of fur farms, reserves and zoos and family members.	
7.3	Visualization methods.<br>Ultrasound scanning of the liver with a portable device - Chison SonoBook6 Portable Ultrasound Diagnostic System	**Ultrasound semiotics CE /AE:**<br>1. cystic formation;<br>2. capsule - not visualized, clearly visualized, multi-layered, calcified;<br>3. shape - round, oval, irregular;<br>4. size - small - up to 5 cm, medium - 5-10 cm, large - more than 10 cm;<br>5. content - homogeneous, heterogeneous (depending on the state of the parasite).<br>**US classification of CE stages:**<br>1. CL (cystic lesion) - cystic formation;<br>2. CE1 and CE2 (active) - active stages, cysts with the presence of viable parasites;<br>3. CE 3a and CE3b (transitional) - transitional, intermediate;<br>4. CE4 and CE5 (inactive) - inactive stages, cysts with the presence of non-viable parasites.<br>**Ultrasound classification AE:**<br>• 1st type - city-shaped, in the form of heterogeneous echogenicity, areas with uneven contours and visible scattered hyperechoic areas, calcifications may be visible;<br>• Type 2 is a pseudocystic pattern with an irregular, hyperechoic rim that is not vascularized on power Doppler;<br>• Type 3 - metastasis-like picture;<br>• Type 4 - hemangioma-like picture;<br>• Type 5 - picture of ossification with signs of calcification.	
7.5	Management of ultrasound positive cases of CE	• Prescription of albendazole - CE1, CE2, CE 3a and CE3b<br>Taking albendazole 1-3 courses at a dose of 10-15 mg/kg of body weight for weight up to 60 kg and for weight over 60 kg, prescribe 400 mg 2 times a day during meals (rich fatty foods) for 28 days with a break for ultrasound control<br>• Observation - CE4 and CE5 (ultrasound once a year)	
9	Risk management and critical points	1) Chronic liver failure;<br>2) Chronic renal failure;<br>3) Allergic reaction.	
	Safety evaluation	1. Allergic reaction: rash on the skin and mucous membranes, itching, redness of the skin; measures - interrupt ABZ, desensitizing therapy, observation, withdrawal of ABZ	

		treatment;<br>2. Chronic liver failure: clinically - jaundice, ascites, impaired consciousness; measures - laboratory monitoring of bilirubin, AST, ALT blood 1 time per month during courses of taking ABZ; Ultrasound of the hepatobiliary system 1 time.	
	Efficiency evaluation	*Primary end result:*<br>1) Ultrasound - cystic single or multiple formations in the liver; Physical examination - hepatomegaly; palpable tumor-like formation in the upper abdominal cavity (standing, sitting, lying on the back and on the right side).<br>*Secondary end result:*<br>1. Clinically - pain in the right hypochondrium;<br>2. Complications of the underlying disease:<br>pain of varying intensity in the right hypochondrium / abdomen; increase in body temperature up to 40 - 41 ° C; chills, profuse sweats; tachycardia; yellowness of the skin and visible mucous membranes; skin itching, urticaria (with a rupture of an echinococcal cyst).	
	Estimation of critical points	Intolerance to ABZ;<br>The complexity of project management due to the participation of several co executors;<br>Difficulties in recruiting and monitoring due to travel to the regions;<br>Severity of the patient's condition due to comorbidity (hepatitis, cirrhosis, HCC) refusal to participate or death;<br>Untimely funding.	
10	IHC IL17 and IL23 analysis in patients with AE and CE after completion of each course of ABZ	The cell suspension, previously labeled with fluorescent dyes (or fluorescent monoclonal antibodies), enters the fluid flow passing through the flow cell. The conditions are chosen in such a way that the cells line up one after another due to the hydrodynamic focusing of the jet in the flow. Forward scatter (used to determine the size of cells) side light scattering (allows to judge the heterogeneity and granularity of cells) fluorescence intensity in 3 fluorescence channels (FL1-FL3).	
11	Molecular study of the proteoscolex of the parasite, determination of the genotype and haplotype	Extraction of DNA from the obtained material using the GeneJET Genomic DNA Purification Kit (ThermoFisher). Add 400 µl of lysis buffer and 5 µl of Proteinase K tothe biomaterial, mix thoroughly by vortexing or pipetting to obtain a homogeneous suspension. Incubate the sample at 56°C with occasional shaking, or use a shaking water bath, shaking platform, or thermomixer until complete cell lysis (3 hours). Add 200 µl of ethanol (96-100%) and mix by pipetting or vortexing. Transfer the prepared lysate to a GeneJET genomic DNA purification column inserted in the collection tube. Centrifuge the column for 1 min at 6000 rpm. Discard the collection tube containing the flow solution. Place the GeneJET Genomic DNA Purification Column in a new 2 ml collection tube. Add 500 µl wash buffer I (with ethanol added). Centrifuge for 1 min at 8000 rpm. Discard the flow and place the purge column back	

		into the collection tube. Add 500 µl Wash Buffer II (with ethanol added) to the GeneJET genomic DNA purification column. Centrifuge for 3 min at maximum speed (≥12000 rpm). Discard the collection tube containing the flow solution and transfer the GeneJET Genomic DNA Purification Column to a sterile 1.5 ml microcentrifuge tube. Add 50 µl of elution buffer to the center of the membrane of the GeneJET genomic DNA purification column to elute the genomic DNA. Incubate for 2 min at room temperature and centrifuge for 3 min at 8000 rpm. Discard the purge column. Use the purified DNA immediately in subsequent studies or store at -20°C.<br>**PCR** will be performed in a 25 µl reaction mixture containing 10x Taq buffer with (NH4)2SO4, 2.5 mM MgCl2, 1 U Taq DNA polymerase, and 200 µM dNTP (Thermo Scientific, Carlsbad, CA, USA), 10 pmol each primer and 20 ng of extracted adult trematode DNA as a template. Thermal reactions were carried out for 35 cycles of denaturation (94°C, 60 s), annealing (50°C, 60 s), and elongation (72°C, 60 s). The resulting restriction fragments were separated by electrophoresis on ethidium bromide containing 1.5% agarose gel using 1x TAE buffer.DNA obtained from Echinococcus cyst was used as a template for amplification of mitochondrial genes.<br>**Sequencing and phylogenetic analysis**<br>The PCR-amplified target gene fragment will be purified using the Quick PCR Purification Kit (Invitrogen, Lithuania) according to the manufacturer's protocols. Sequencing is carried out in accordance with the instructions for the Seq Studio Genetic Analyzer (Thermo Fisher Scientific Applied Biosystems). The resulting nucleotide sequences are visually analyzed using Bio Capt version 11.0.<br>Multiple alignment of the obtained sequences using the ClustalW algorithm in the MEGA program (v.11). Export alignments as a NEXUS extension and use as input to TCS v1.21 for haplotype identification, network building, and diversity index estimation. Phylogenetic analysis is built according to the nearest neighbor (NJ) algorithm using MEGA v.11 software	
12	Statistical	SPSS Statistical Package, version 15.0.	

**Социологический опрос**						
ФИО						
Дата рождения			Возраст			
Пол	муж		жен			
Адрес проживания						
Уровень образования	Университет		Колледж		Школа	
Профессия						
**Сведения о факторах риска проживание**						
В эндемичном регионе?	Жамбылская	Туркестанская	Алматинская	Жетысуская		
Точный адрес проживания в эндемичной регионе?						
Годы проживания в эндемичном регионе?	________год	________год				
Сколько лет (месяцев) проживал в эндемичном регионе?						
**Контакт с собакой**						
Есть ли дворовая собака?	Да	Нет				
Вашей собаке в течение последних 3-5 летпроводили противоглистную терапию?	Да	Нет				
Содержите при-отарная, служебная собака?	Да	Нет				
Собака на привязи?	Да	Нет				
Собаку отпускаете (убегает со двора?	Да	Нет				
Собаку вы сами кормите?	Да	Нет				
Собаку кормите сырыми внутренними органами животных?	Да	Нет				
ВЫ убираете за собакой?	Да	Нет				
**Контакт с сельскохозяйственными животными**						
ВЫ употребляете немытые ягоды (малина, клубника, яблоки и др. фрукты с земли)?	Да	Нет				
У вас есть привычка брать в рот и надкусывать дикорастущие травы?	Да	Нет				
**Ваш род деятельности в течение жизни связан?**						
Работник животноводческой фермы?	Да	Нет				
Работник мясокомбината?	Да	Нет				
Работник убойного цеха?	Да	Нет				
Работник цеха утилизации и захоронения животных?	Да	Нет				
Разделка и продажа мяса и внутренних органов?	Да	Нет				
Работаете чабаном, охотником, егерем?	Да	Нет				
Работа в мастерской по переработке мехового сырья?	Да	Нет				
Работаете ветеринаром?	Да	Нет				
Работник фермы, заповедников и зоопарков?	Да	Нет				
**Ваш член семьи (муж, сын, дочка и др.) работает?**						
Работник животноводческой фермы?	Да	Нет				
Работник мясокомбината?	Да	Нет				
Работник убойного цеха?	Да	Нет				
Работник цеха утилизации и захоронения животных?	Да	Нет				
Разделка и продажа мяса и внутренних органов?	Да	Нет				
Работаете чабаном, охотником, егерем?	Да	Нет				
Работник фермы, заповедников и зоопарков?	Да	Нет				

Социологиялық сауалнама

Толықаты					
Туғанкүні			Жасы		
Жынысы		Еркек		Әйел	
Тұрғынүймекен-жайы					
Білімдеңгейі	Университет		Колледж		Мектеб
Мамандық					

Тәуекел факторларын орн аластыру туралы ақпарат

Сіз қай эндемикалық аймақта тұрасыз?	Жамбыл	Түркістан	Алматы	Жетісу
Эндемиялықаймақтатұратыннақтымекенжай?				
Эндемиялықаймақтатұружылдары?	_________жыл	________жыл		
Сізэндемиялықаймақтақаншажыл (ай) тұрдыңыз?				

Итпен байланыс

Сіздіңауладаит бар ма?	Иә	Жоқ
Сіздіңитіңізгесоңғы 3-5 жылдынішіндеқұртқақарсыдәріберілдіма?	Иә	Жоқ
Сіз отар немесеқызметтікитұстайсыз ба?	Иә	Жоқ
Ит байлаулыма?	Иә	Жоқ
Сізитіңіздісыртқашығарасыз ба немесеауладанқашыпкетеді ма?	Иә	Жоқ
Сізитке өзіңізтамақ бересіз ба?	Иә	Жоқ
Сізитіңізгежануарлардыңшикімүшелерін бересіз ба?	Иә	Жоқ
Сіз итіңіздіңартынантазалайсыз ба?	Иә	Жоқ

Ауылшаруашылығыжануарларыменбайланыс

СІЗ жуылмағанжидектердітаңқурай, құлпынай, алмажәнежердегібасқажемістер жумайжейсіз бе?	Иә	Жоқ
Жабайышөптердіаузыңызғасалып, тістепалатынәдетіңіз бар ма?	Иә	Жоқ

Сіздінкәсібіңізбайланыстыма?

Мал фермасыныңжұмысшысыз ба?	Иә	Жоқ
Еткомбинатыныңжұмысшысыз ба?	Иә	Жоқ
Қасапхананыңжұмысшысыз ба?	Иә	Жоқ
Жануарлардыкәдегежаратужәнекөмуқызметкерісіз?	Иә	Жоқ
Ет пен ішкіағзалардыкесужәне сатуға қатысасыз ба?	Иә	Жоқ
Сізшопан, аңшыболыпжұмысістейсіз бе?	Иә	Жоқ
Теріөңдеуцехындажұмысістейсіз бе?	Иә	Жоқ
Сізветеринарсыз ба?	Иә	Жоқ
Ферма, қорықжәнезообаққызметкерісіз ба?	Иә	Жоқ

Отбасыңыздыңмүшесі (күйеуі, ұлы, қызы, т.б.) жұмысістей ме?

Мал фермасыныңжұмысшысы?	Иә	Жоқ
Еткомбинатыныңжұмысшысы?	Иә	Жоқ
Қасапханажұмысшысыма?	Иә	Жоқ
Жануарлардыкәдегежаратужәнекөмуқызметкері?	Иә	Жоқ
Ет пен ішкіағзалардыкесужәне сату?	Иә	Жоқ
Шопан, аңшыболыпжұмысістейді ма?	Иә	Жоқ
Ферма, қорықжәнезообаққызметкері ма?	Да	Нет

ИНФОРМИРОВАННОЕ СОГЛАСИЕ

Письменное добровольное согласие пациента при инвазивных вмешательствах

Приказ Министра здравоохранения и социального развития Республики Казахстан от 20 мая 2015 года № 364. «Об утверждении формы письменного добровольного согласия пациента при инвазивных вмешательствах»

Я, (подчеркнуть) пациент/законный представитель:

____,

(фамилия, имя, отчество (при его наличии) пациента/законного представителя) находясь в ____

(наименование медицинской организации)

даю свое согласие на проведение (подчеркнуть): мне/лицу, законным представителем которого я являюсь: ____

____

(фамилия, имя, отчество (при его наличии), дата рождения) следующей процедуры: ____

(указать наименование процедуры)

1. Я информирован/(а) о целях, характере, неблагоприятных эффектах планируемого инвазивного вмешательства, согласен(на) со всеми подготовительными и сопровождающими возможными анестезиологическими мероприятиями, а также с необходимыми побочными вмешательствами. Я предупрежден(а), что во время инвазивного вмешательства могут возникнуть непредвиденные обстоятельства, риски и осложнения и понимаю, что это может включать нарушения со стороны сердечно-сосудистой, нервной, дыхательной и других систем жизнедеятельности организма, что это связано с непреднамеренным причинением вреда здоровью. В таком случае, я согласен(на) на применение врачами всевозможных методов лечения, направленных на устранение вышеуказанных осложнений.

2. Я ставлю в известность врача обо всех проблемах, связанных с моим здоровьем (со здоровьем лица, законным представителем которого я являюсь):
наследственность;
аллергические проявления;
индивидуальная непереносимость лекарственных препаратов и продуктов питания;
потребление табачных изделий;
злоупотребление алкоголем;
злоупотребление наркотическими средствами; перенесенные травмы, операции, заболевания, анестезиологические пособия; экологические и производственные факторы физической, химической или биологической природы, воздействующие на меня (лица, законным представителем которого я являюсь) во время жизнедеятельности; принимаемые лекарственные средства.

3. Я имел(а) возможность задавать любые вопросы врачу и на все вопросы получил(а) исчерпывающие ответы.

4. Я ознакомлен(а) со всеми пунктами настоящего документа и согласен(а) с ними.

Подпись заявителя: ___________ __________(пациент/законный представитель)

Дата заполнения: ______/__________________/20__ __ г.

Врач: _________________________(фамилия, имя, отчество (при его наличии)

Подпись: ___________________

Примечание:

1. Инвазивное вмешательство – медицинская процедура, связанная с проникновением через естественные внешние барьеры организма (кожа, слизистые оболочки) (инъекция, диагностическая процедура, хирургическая операция и так далее).

2. Согласие на проведение инвазивного вмешательства может быть отозвано, за исключением случаев, когда медицинские работники по жизненным показаниям уже приступили к инвазивному вмешательству и его прекращение или возврат невозможны в связи с угрозой для жизни и здоровья пациента.

ИНФОРМАЦИОННЫЙ ЛИСТ

1.1. Наименование темы проекта «Разработка молекулярно-эпидемиологической карты распространенности эхинококкоза в человеческой популяции Казахстана»

1.2. Руководитель проекта – к.м.н., профессор Исмаилова Гульзия Нуртазаевна

1.3. Наименование приоритетного направления развития науки, по которому подается заявка - 6. Наука о жизни и здоровье.

1.4. Наименование специализированного научного направления, по которому подается заявка, область и вид исследований - 6.3 Инновационные исследования в медицине и общественном здравоохранении. Область исследования по Межгосударственный рубрикатор научно-технической информации: 76 - Медицина и здравоохранение: (76.29.62 - Рентгенология и медицинская радиология,76.29.00 - Клиническая медицина); 76.00.00 - Медицина и здравоохранение. Вид исследования: прикладное исследование.

1.5. Дата начала и завершения проекта, его продолжительность в месяцах: февраль 2023года – декабрь 2025 года, 35 месяцев.

1.6. Финансирование – Комитет науки Министерства науки и высшего образования Республики Казахстан

Исследование имеет цель изучение генотипической распространенности и оценки эффективности медикаментозного лечения эхинококкоза в человеческой популяции Казахстана, включая эндемичные регионы, для эффективного реагирования, управления и снижения бремени кистозного и мультифокального эхинококкоза печени методом ультразвукового сканирования в рамках глобальной дорожной карты ВОЗ «Neglected tropical diseases» 2021–2030.

В рамках проекта планируется стратификация 21 населенных пунктов, относительно численности проживающего детского и взрослого населения около 2000 человек в возрасте от 5 до 70 лет, занимающихся животноводством или имеющих сельскохозяйственных животных в дворовом хозяйстве. Отбор по 3села (аула, поселка) в каждом из 7 регионов, в которых в течение последних 5 лет, не проводился массовый скрининг эхинококкоза. Включая эндемичные (Туркестанской, Жамбылской и Алматинской / Жетысуской)и центральные регионы (Акмолинская, Карагандинская и Костанайская) Казахстана, для анализа заболеваемости эхинококкозом, относительно генотипической характеристика паразита в человеческой популяции.

В отобранных населенных пунктах методом анкетирования опросником местных жителей, будут изучены факторы риска, способствующие высокой заболеваемости эхинококкозом: alveolar echinococcosis (AE) и cystic echinococcosis (CE), для научного обоснования национальной скрининговой программы выявления эхинококкоза печени в эндемичных зонах Казахстана.

Будет усовершенствован алгоритм диагностики эхинококкоза печени. Будет внедрена УЗИ классификация АЕ, на основе УЗИ семиотики альвеолярного эхинококкоза печени, основаная на 5 типах поражения печени.

Будут выполняться выезды в регионы, встречи с населением, наглядная презентация, разъяснительная беседа в формате вопрос-ответ по эхинококкозу: о значении гигиены рук, о других мерах безопасности, о противопаразитарной терапии 2-4 раза в год (празиквантел и др) дворовых и отарных собак.

Будет проводиться УЗИ печени взрослого и детского населения портативным аппаратом, идентификация кист печени согласно УЗИ классификации СЕ и АЕ.

Тактика ведения пациентов, согласно протоколам МЗ РК. Пациентам с УЗИ характеристикой эхинококкового поражения печени, на месте будет выполнен забор биологического образца (кровь из локтевой вены), для транспортировки сыворотки в термо контейнере в лабораторию Алматы для иммунологического анализа антител класса IgG к антигенам эхинококкоза и суммарных антител класса IgE.

По результатам УЗИ печени, пациентам с СЕ1-СЕ3b стадиями СЕ на месте консультация хирурга назначение альбендазола (ABZ) на курс лечения ABZ 400 мг в форме таблетки 1-2 упаковки (относительно веса пациента) на первый курс лечения бесплатно. Контрольное УЗИ через 1 месяц. По результатам УЗИ контроля 2 и 3 курсы лечения бесплатно ABZ 400 мг. Предупредить пациента о приеме ABZ во время еды (обильной жирной пищи) и о возможных побочных эффектах, сообщить по контактному номеру телефона горячей линии (+77751808285), в случаях приема ABZ.

Будет изучена активность интерлейкинов 17 и 23 у пациентов CE / AE после курсов приема ABZ, как индикатор успешной элиминации паразита.

Пациенты с кистозным эхинококкозом меньше 5 см, после первого курса ABZ, будут направлены для дальнейшего лечения ABZ + PAIR, а больше 5 см ABZ + вмешательство, в ННЦХ им. А.Сызганова по квоте.

Будет создан геномный банк E.granulosus и E.multilocularis из человеческих изолятов для молекулярной идентификации фенотипов и гаплотипов E. granulosus и E. multilocularis с периодом охвата 2023–2025 годы. Будет изучена молекулярная эпидемиология человеческого эхинококкоза в Казахстане, для определения путей распространения и причин неуклонного роста заболеваемости эхинококкозом населения Казахстана.

Утверждение в Локальной комиссии по биоэтике ВШМ КазНУ Аль-Фараби, анкеты-опросника, информационного листа и форм информированного согласия для участия в данном исследовании и предоставления биологического материала для научного исследования. Работа на уровне акиматов: разъяснение цели скрининга, подготовка помещения (клуб, медпункт и др.), уведомление населения о планируемом исследовании.

АҚПАРАТТЫ КЕЛІСІМ

Науқастың инвазиялық араласуға жазбаша ерікті келісімі

«Инвазиялық араласулар кезінде пациенттің ерікті түрдегі жазбаша келісімінің нысанын бекіту туралы» Қазақстан Республикасы Денсаулық сақтау және әлеуметтік даму министрінің 2015 жылғы 20 мамырдағы № 364 бұйрығы.

Мен, (астын сызу) пациент/заңды өкіл:

____,

(пациенттің/заңды өкілдің тегі, аты, әкесінің аты (бар болса))

____

(медициналық ұйымның атауы)

орындауға келісім беремін (астын сызу): мені/заңды өкілі болып табылатын тұлғаны:

____

____

(тегі, аты, әкесінің аты (бар болса), туған күні) мынадай тәртіппен:

____

(процедураның атауын көрсетіңіз)

1. Мен жоспарланған инвазивті араласудың мақсаттары, сипаты, жағымсыз әсерлері туралы хабардар етемін, барлық дайындық және ілеспе мүмкін болатын анестетикалық шаралармен, сондай-ақ қажетті жанама араласулармен келісемін. Маған инвазивті араласу кезінде күтпеген жағдайлар, қауіптер мен асқынулар туындауы мүмкін екендігі ескертілді және бұл жүрек-тамыр, жүйке, тыныс алу және басқа да ағзаның өмірлік маңызды жүйелерінің бұзылуын қамтуы мүмкін екенін түсінемін, бұл денсаулыққа байқаусызда зиян келтірумен байланысты. Бұл жағдайда мен дәрігерлердің жоғарыда аталған асқынуларды жоюға бағытталған емдеудің барлық мүмкін әдістерін қолдануына келісемін.

2. Мен өзімнің денсаулығыма байланысты барлық мәселелер туралы дәрігерге хабарлаймын (мен заңды өкілі болып табылатын адамның денсаулығы):

тұқым қуалаушылық;

аллергиялық реакциялар;

есірткіге және тағамға жеке төзбеушілік;

темекі өнімдерін тұтыну;

алкогольді теріс пайдалану;

есірткіні теріс пайдалану; өткен жарақаттар, операциялар, аурулар, анестетикалық араласулар; менің өмірімде маған (мен заңды өкілі болып табылатын адамға) әсер ететін физикалық, химиялық немесе биологиялық сипаттағы экологиялық және өндірістік факторлар; қабылданған дәрілер.

3. Дәрігерге кез келген сұрақ қою мүмкіндігіне ие болдым және барлық сұрақтарға жан-жақты жауап алдым.

4. Мен бұл құжаттың барлық тармақтарымен таныспын және олармен келісемін.

Өтініш берушінің қолы: ___________ __________(пациент/заңды өкілі)

Аяқталған күні: ______/__________________/20___

Дәрігер: ________________________(тегі, аты, әкесінің аты (бар болса)
Қолы: ____________________

Ескерту:

1. Инвазиялық араласу – бұл ағзаның табиғи сыртқы кедергілері (тері, шырышты қабаттар) арқылы енуді көздейтін медициналық процедура (инъекция, диагностикалық процедура, хирургиялық араласу және т.б.).

2. Медицина қызметкерлері өмірлік маңызды көрсеткіштер бойынша инвазиялық араласуды бастаған және пациенттің өмірі мен денсаулығына қауіп төнгендіктен оны тоқтату немесе қайтару мүмкін болмаған жағдайларды қоспағанда, инвазиялық араласуды жүргізуге келісімнің күші жойылуы мүмкін.

АҚПАРАТ ПАРАҒЫ

1.1. Жоба тақырыбының тақырыбы «Қазақстан халқының эхинококкозының таралуының молекулярлық-эпидемиологиялық картасын жасау»

1.2. Жоба жетекшісі – PhD докторы, профессор Исмаилова Гүлзия Нұртазақызы

1.3. Өтінім берілетін ғылыми дамудың басым бағытының атауы – 6. Өмір және денсаулық ғылымы.

1.4. Өтінім берілетін мамандандырылған ғылыми бағыттың атауы, зерттеу саласы мен түрі – 6.3 Медицина және денсаулық сақтау саласындағы инновациялық зерттеулер. Ғылыми-техникалық ақпараттың мемлекетаралық рубрикаторына сәйкес зерттеу бағыты: 76 – Медицина және денсаулық сақтау: (76.29.62 – Рентгенология және медициналық радиология, 76.29.00 – Клиникалық медицина); 76.00.00 – Медицина және денсаулық сақтау. Зерттеу түрі: қолданбалы зерттеу.

1.5. Жобаның басталу және аяқталу күні, оның айлармен ұзақтығы: 2023 жылғы ақпан – 2025 жылғы желтоқсан, 35 ай.

1.6. Қаржыландыру – Қазақстан Республикасы Ғылым және жоғары білім министрлігінің Ғылым комитеті

Зерттеу ДДҰ-ның жаһандық жол картасы аясында ультрадыбыстық сканерлеуді қолдана отырып, бауырдың кистозды және көп ошақты эхинококкозына тиімді әрекет ету, басқару және ауыртпалығын азайту үшін Қазақстан популяциясында, оның ішінде эндемиялық аймақтарда эхинококкоздың генотиптік таралуын зерттеу және дәрілік емнің тиімділігін бағалауға бағытталған. 2021–2030 жж.

Жоба мал шаруашылығымен айналысатын немесе ауласында ауылшаруашылық малдары бар 5 пен 70 жас аралығындағы 2000-ға жуық адам тұратын балалар мен ересектердің санына қатысты 21 елді мекенді стратификациялауды жоспарлап отыр. Соңғы 5 жылда эхинококкозға жаппай скринингтік тексеру жүргізілмеген 7 облыстың әрқайсысында 3 ауылды (ауыл, елді мекен) таңдау. Соның ішінде Қазақстанның эндемикалық (Түркістан, Жамбыл және Алматы/Жетісу) және орталық облыстары (Ақмола, Қарағанды және Қостанай) адам популяциясындағы паразиттің генотиптік ерекшеліктеріне қатысты эхинококкозбен аурушаңдығын талдау үшін. Таңдалған елді мекендерде жергілікті тұрғындардың сауалнамалық сауалнамасын пайдалана отырып, Қазақстан аумағындағы бауыр эхинококкозын анықтау бойынша ұлттық скринингтік бағдарламаны ғылыми негіздеу үшін эхинококкоздың жоғары сырқаттануына ықпал ететін қауіп факторлары: альвеолярлы эхинококкоз (АЭ) және кисталық эхинококкоз (КЭ) зерттелетін болады. Бауыр эхинококкозын диагностикалау алгоритмі жетілдірілетін болады. Бауырдың альвеолярлы эхинококкозының ультрадыбыстық семиотикасы негізінде, бауыр зақымдануының 5 түріне негізделген АЭ ультрадыбыстық классификациясы енгізіледі.

Өңірлерге сапарлар, халықпен кездесулер, көрнекі презентациялар, эхинококкоз бойынша сұрақ-жауап форматында түсіндірме әңгімелер жүргізіледі: қол гигиенасын сақтаудың маңыздылығы туралы, басқа да қауіпсіздік шаралары туралы, аула және отар иттеріне жылына 2-4 рет паразиттерге қарсы терапия (празиквантел және т.б.) туралы.

Ересектер мен балалардың бауырының ультрадыбыстық зерттеуі портативті аппаратпен жүргізіледі, КЭ және АЭ ультрадыбыстық классификациясы бойынша бауыр кисталарын анықтау.

Қазақстан Республикасы Денсаулық сақтау министрлігінің хаттамалары бойынша пациентті жүргізу тактикасы. Эхинококкозбен бауыр зақымдануының ультрадыбыстық сипаттамасы бар науқастар үшін термиялық контейнердегі сарысуды эхинококкоз антигендеріне және жалпы IgE

антиденелеріне иммунологиялық талдау үшін Алматы зертханасына тасымалдау үшін биологиялық үлгі (кубитальды венадан қан) алынады.

Бауырдың ультрадыбыстық зерттеуінің нәтижелері бойынша CE1-CE3b сатылары бар науқастарға хирургтың кеңесі және таблетка түріндегі 400 мг ABZ, бірінші емдеу курсына 1-2 қаптама (пациент салмағына қатысты) тегін емдеу курсы үшін альбендазол (ABZ) тағайындалады. 1 айдан кейін ультрадыбысты бақылау. Ультрадыбыстық бақылау нәтижелері бойынша емдеудің 2 және 3 курстары ABZ 400 мг тегін. Науқасты тамақ кезінде ABZ қабылдау (майлы тағамдар) және ықтимал жанама әсерлер туралы ескертіңіз, ABZ қабылдаған жағдайларда сенім телефонына (+77751808285) хабарласыңыз.

17 және 23 интерлейкиндердің белсенділігі паразиттердің сәтті жойылуының көрсеткіші ретінде ABZ курстарынан кейін КЭ / АЭ пациенттерінде зерттелетін болады.

Кистозды эхинококкозбен ауыратындар 5 см-ден аз, ABZ бірінші курсынан кейін ABZ + PAIR, ал 5см-ден жоғары ABZ +интервенциямен әрі қарай емдеуге А.Сызғанов атындағы Ұлттық ғылыми хирургия орталығына квота бойынша жіберіледі.

E. granulosus және E. multilocularis фенотиптері мен гаплотиптерін молекулярлық сәйкестендіру үшін адам изоляттарынан E. granulosus және E. multilocularis геномдық банкі құрылады, қамту мерзімі 2023-2025 жж. Қазақстан халқының эхинококкоз ауруының таралу жолдарын және тұрақты өсу себептерін анықтау үшін Қазақстандағы адам эхинококкозының молекулярлық эпидемиологиясы зерттелетін болады.

Әл-Фараби атындағы ҚазҰУ Жоғары Басқару мектебінің Жергілікті биоэтика комиссиясының сауалнаманы, ақпараттық парақты және осы зерттеуге қатысуға ақпараттандырылған келісім нысандарын бекіту және биологиялық ғылыми зерттеуге арналған материал. Әкімдіктер деңгейінде жұмыс: скрининг мақсатын түсіндіру, үй-жайларды (клуб, медициналық пункт және т.б.) дайындау, халықты жоспарланған зерттеу туралы хабардар ету.

Согласие родителей (законных представителей) на медицинское вмешательство

Я, ____

(ФИО родителя (законного представителя)

Законный представитель (мать, отец, опекун, попечитель) несовершеннолетнего

(нужное подчеркнуть)

____

(ФИО несовершеннолетнего)

В соответствии *сп.18ст.77 Кодекса Республики Казахстан «О здоровье народа и системе здравоохранения» от 7 июля 2020 года № 360 - VI ЗРК* даю информированное согласие для участия моего ребенка в проекте АР19676676 «Разработка молекулярно-эпидемиологической карты распространенности эхинококкоза в человеческой популяции Казахстана» в качестве пациента для обследования и лечения, а именно: оказание медицинской помощи, выполнение исследований, вмешательств, ультразвуковых и иммунологических методов исследования по медицинским показаниям, согласно действующим клиническим протоколам Министерства здравоохранения Республики Казахстан.

Я даю согласие на проведение при необходимости следующих вмешательств:

- Стандартизированное анкетирование
- Медицинский осмотр;
- Ультразвуковое исследование органов брюшной полости;
- Информирование медицинских сотрудников проекта, а также медицинских поликлиники, педагогов, о состоянии здоровья ребенка;
- Систематический контроль за состоянием здоровья ребенка, в активном выявление побочных явлений;
- Контроль за соблюдением правил личной гигиены ребенком;
- При необходимости оказывать амбулаторно-поликлиническую помощь.
- При несчастных случаях–оказание неотложной медицинской помощи, госпитализация по медицинским показаниям детей в лечебно-профилактическое учреждение.

В случае угрозы жизни и здоровью ребенка необходимо обратиться за консультационной и лечебной помощью в близлежащие медицинские учреждения.

В доступной форме мне разъяснены цели, методы исследования, связанный сними риск, возможные варианты медицинских вмешательств, их последствия, в том числе вероятность развития осложнений, возможность направления ребенка на лечение в лечебно-профилактическое учреждение, а также предполагаемые результаты.

Я ознакомлен (ознакомлена) и согласен (согласна) со всеми пунктами настоящего документа, положения которого мне разъяснены, мною поняты.

Настоящее согласие дано мной «_______»_____________20г. и действует на время обследования и лечения моего ребенка / опекуна.

Я оставляю за собой право отозвать свое согласие посредством составления соответствующего письменного документа, который может быть направлен мной в Казахский национальный университет им. аль-Фараби, по адресу Казахстан, Алматы, 050040 пр. Аль-Фараби, 71, Тел. +7 (727) 377-33-30, Факс +7 (727) 377-33-44, info@kaznu.edu.kz по почте заказным письмом с уведомлением о вручении либо по электронной почте.

Законный представитель:

___________________/ ____

Подпись *ФИО*

МЕДИЦИНАЛЫҚ АРАЛАСУҒА АТА-АНАНЫҢ (ЗАҢДЫ ӨКІЛДЕРДІҢ) КЕЛІСІМІ

Мен, ____

(Ата-анасының (заңды өкілінің) Т.А.Ә.)

Кәмелетке толмаған баланың заңды өкілі (анасы, әкесі, қорғаншысы, қамқоршысы).

(сәйкесінше астын сызу)

____

(Кәмелетке толмаған баланың аты-жөні)

Қазақстан Республикасының 2020 жылғы 7 шілдедегі № 360 – VI ЗРК «Халық денсаулығы және денсаулық сақтау жүйесі туралы» Кодексінің 77-бабының 18-тармағына сәйкес баламның АП19676676 «Қазақстандағы адамикококккок предметі популяциясының молекулярлық эпидемиологиялық картасын әзірлеу» жобасына қатысуға келісімін беремін. тексеру және емдеу, атап айтқанда: Қазақстан Республикасы Денсаулық сақтау министрлігінің қолданыстағы клиникалық хаттамаларына сәйкес медициналық көмек көрсету, медициналық көрсеткіштер бойынша зерттеулер, араласулар, ультрадыбыстық және иммунологиялық зерттеу әдістерін орындау.

Қажет болған жағдайда келесі араласуларға келісім беремін:

- Стандартталған сауалнама
- медициналық тексеру;
- құрсақ қуысы мүшелерінің ультрадыбыстық зерттеуі;
- жобаның медициналық қызметкерлерін, сонымен қатар дәрігерлік амбулаторияны, мұғалімдерді баланың денсаулығы туралы хабардар ету;
- Баланың денсаулығын жүйелі бақылау, жанама әсерлерді белсенді түрде анықтау;
- баланың жеке гигиена ережелерін сақтауын қадағалау;
- Қажет болған жағдайда амбулаторлық-емханалық көмек көрсетіңіз.
- жазатайым оқиғалар кезінде – шұғыл медициналық көмек көрсету, балаларды емдеу-алдын алу мекемесіне медициналық себептер бойынша жатқызу.

Баланың өмірі мен денсаулығына қауіп төнген жағдайда жақын маңдағы медициналық мекемелерге кеңес алып, медициналық көмекке жүгіну қажет.

Маған қолжетімді нысанда мақсаттар, зерттеу әдістері, ілеспе қауіп, медициналық араласулардың ықтимал нұсқалары, олардың салдары, оның ішінде асқыну ықтималдығы, баланы емдеу-алдын алу мекемесіне емдеуге жіберу мүмкіндігі, сондай-ақ күтілетін нәтижелер түсіндірілді.

Мен осы құжаттың барлық тармақтарын оқыдым және келісемін, оның ережелері маған түсіндірілді, мен түсінемін.

Бұл келісімді мен «_______» 20 бердім және баламды/қамқоршымды тексеру және емдеу кезеңі үшін жарамды.

Мен Қазақстан, Алматы, 050040 Әл-Фараби даңғылы, 71, тел. мекенжайы бойынша Әл-Фараби атындағы Қазақ ұлттық университетіне жіберуге болатын тиісті жазбаша құжатты ресімдеу арқылы келісімімді қайтарып алу құқығын өзіме қалдырамын. +7 (727) 377-33-30, факс +7 (727) 377-33-44, info@kaznu.edu.kz алғаны туралы хабарламасы бар тапсырыс хат немесе электрондық пошта арқылы.

Заңды өкілі:

_____________________ / ____

Қолы *Толық аты-жөні*
